# Supplementary material for: High-Frequency Imaging Reveals Synchronised Delta- and Theta-Band Ca2+ Oscillations in the Astrocytic Soma In Vivo
Source: Int J Mol Sci. 2024 Aug 16;25(16):8911. doi: 10.3390/ijms25168911 (PMC11354863; doi:10.3390/ijms25168911)
Supplement: Supplementary file 1 [file ijms-25-08911-s001.zip › ijms-3128143-supplementary.pdf]

Supplementary information

High frequency imaging reveals delta and theta band Ca^{2+} oscillations in the astrocytic soma

Márton Péter [1,2] and László Héja [1,*]

[1] Institute of Organic Chemistry, HUN-REN Research Centre for Natural Sciences, Magyar tudósok körútja 2, 1117 Budapest, Hungary; peter.marton@ttk.hu
[2] Hevesy György PhD School of Chemistry, ELTE Eötvös Loránd University, 1117 Budapest, Hungary
* Correspondence: heja.laszlo@ttk.hu; Tel.: +36-1-382-6619

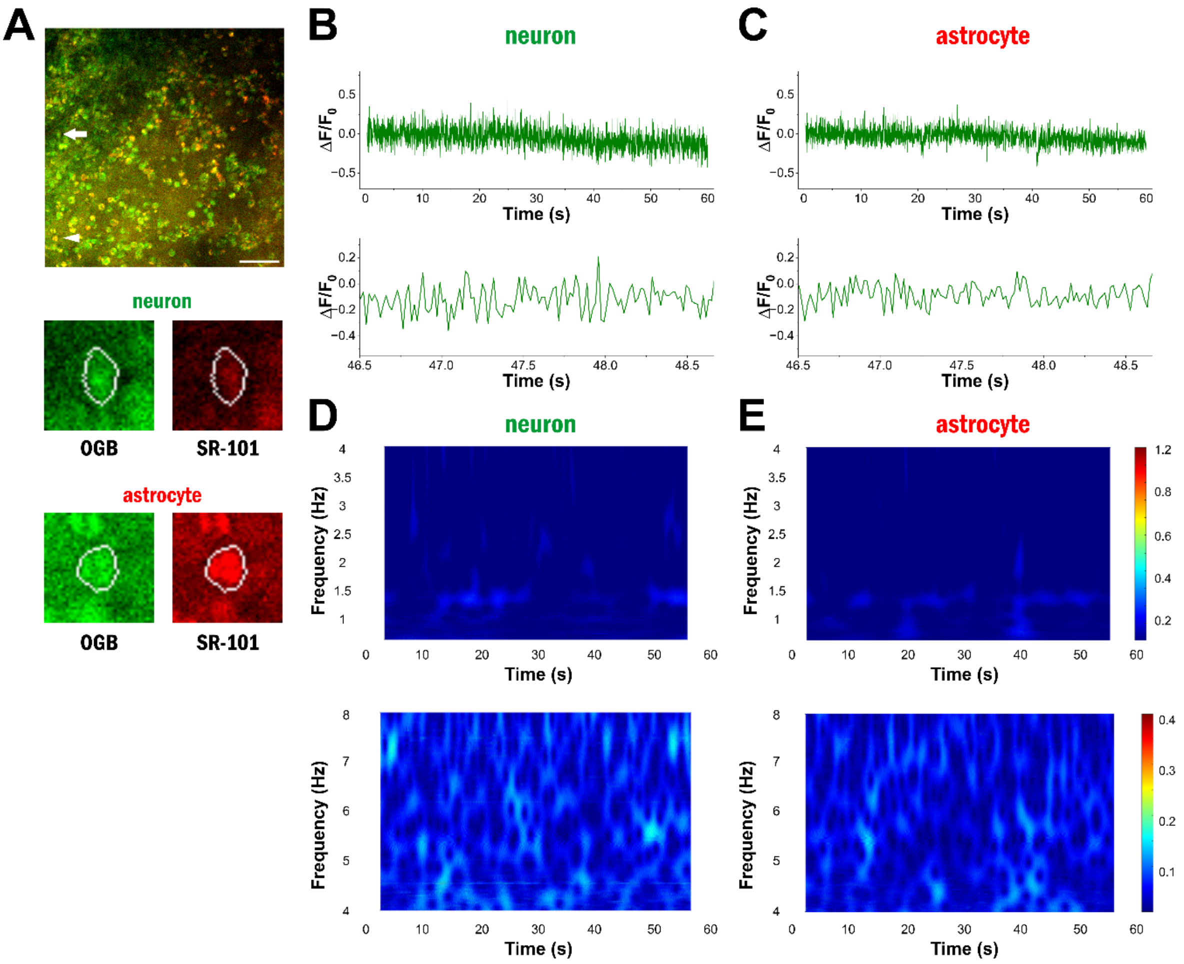

Figure S1. Non-responsive cells during ketamine/xylazine anaesthesia. (A) OGB-1 and SR-101 labelling in layer 2 of the V1 cortex in vivo during ketamine/xylazine anaesthesia. The analysed non-responsive neuron is marked by an arrow, the analysed non-responsive astrocyte is marked by an arrowhead. Boundary of the ROI in which the cell type was determined is shown in white. Scale bar: 100 µm. (B) OGB-1 fluorescence intensity changes of the selected neuron during the 60 s long imaging session (top) and a close-up view of the $\Delta F/F_0$ trace (bottom). (C) OGB-1 fluorescence intensity changes of the selected astrocyte during the 60 s long imaging session (top) and a close-up view of the $\Delta F/F_0$ trace (bottom). (D) Wavelet analysis of the selected neuron in the delta (0.5−4 Hz, top) and the theta (4−8 Hz, bottom) frequency ranges. (E) Wavelet analysis of the selected astrocyte in the delta (0.5−4 Hz, top) and the theta (4−8 Hz, bottom) frequency ranges.
